# Comparison of circulation patterns of mumps virus in the Netherlands and Spain (2015–2020)

**DOI:** 10.3389/fmicb.2023.1207500

**Published:** 2023-06-16

**Authors:** Ana M. Gavilán, Linda van de Nes-Reijnen, Ana Castellanos, Tom Woudenberg, Noemí López-Perea, Josefa Masa-Calles, Juan E. Echevarría, Aurora Fernández-García, Rogier Bodewes

**Affiliations:** ^1^Centro Nacional de Microbiología (CNM), Instituto de Salud Carlos III (ISCIII), Madrid, Spain; ^2^CIBER de Epidemiología y Salud Pública (CIBERESP), ISCIII, Madrid, Spain; ^3^Centre for Infectious Disease Control, National Institute for Public Health and the Environment (RIVM), Bilthoven, Netherlands; ^4^Centro Nacional de Epidemiología, Instituto de Salud Carlos III (ISCIII), Madrid, Spain

**Keywords:** laboratory surveillance, molecular epidemiology, mumps virus, SH sequence, MF-NCR sequence, the Netherlands, Spain

## Abstract

**Background:**

Mumps is a viral infection mainly characterized by inflammation of the parotid glands. Despite of vaccination programs, infections among fully vaccinated populations were reported. The World Health Organization (WHO) recommends molecular surveillance of mumps based on sequencing of the small hydrophobic (SH) gene. The use of hypervariable non-coding regions (NCR) as additional molecular markers was proposed in multiple studies. Circulation of mumps virus (MuV) genotypes and variants in different European countries were described in the literature. From 2010 to 2020, mumps outbreaks caused by genotype G were described. However, this issue has not been analyzed from a wider geographical perspective. In the present study, sequence data from MuV detected in Spain and in The Netherlands during a period of 5  years (2015- March 2020) were analyzed to gain insights in the spatiotemporal spread of MuV at a larger geographical scale than in previous local studies.

**Methods:**

A total of 1,121 SH and 262 NCR between the Matrix and Fusion protein genes (MF-NCR) sequences from both countries were included in this study. Analysis of SH revealed 106 different haplotypes (set of identical sequences).

**Results:**

Of them, seven showing extensive circulation were considered variants. All seven were detected in both countries in coincident temporal periods. A single MF-NCR haplotype was detected in 156 sequences (59.3% of total), and was shared by five of the seven SH variants, as well as three minor MF-NCR haplotypes. All SH variants and MF-NCR haplotypes shared by both countries were detected first in Spain.

**Discussion:**

Our results suggest a transmission way from south to north Europe. The higher incidence rate of mumps in Spain in spite of similar immunization coverage in both countries, could be associated with higher risk of MuV exportation. In conclusion, the present study provided novel insights into the circulation of MuV variants and haplotypes beyond the borders of single countries. In fact, the use of MF-NCR molecular tool allowed to reveal MuV transmission flows between The Netherlands and Spain. Similar studies including other (European) countries are needed to provide a broader view of the data presented in this study.

## 1. Introduction

Mumps is a vaccine-preventable disease caused by mumps virus (MuV), which is pleomorphic and has a single non-segmented negative sense RNA molecule as genome. Seven transcription units are encoded in it: the nucleoprotein (N), the V/phospo-/I proteins (V/P/I), the matrix protein (M), the fusion protein (F), the small hydrophobic protein (SH), the haemagglutinin-neuraminidase protein (HN) and the polymerase (L) (Rubin et al., 2015). MuV belongs to the genus *Orthorubulavirus* of the family *Paramyxoviridae* (ICTV, n.d.).

MuV is transmitted human-to-human by direct contact through respiratory droplets and contaminated fomites. The main symptom is swelling of parotid glands (Lam et al., 2020). In addition, other unspecific symptoms such as fever, headache, malaise or anorexia can occur. Orchitis, mastitis, oophoritis and pancreatitis are less frequent symptoms and encephalitis and aseptic meningitis can be rare complications (Lam et al., 2020). In about a third of unvaccinated individuals and three quarters of vaccinated individuals, the infection with mumps virus does not result in recognized clinical signs (Philip et al., 1995; Dittrich et al., 2011).

Molecular surveillance is a useful tool to study the origin and routes of transmission of pathogens. World Health Organization recommends molecular surveillance of MuV based on sequencing of the SH gene (WHO, 2012). Based on genetic variation of the SH and HN gene sequence, 12 genotypes were identified: A, B, C (including former genotype E), D, F, G, H, I, J, K (including former genotype M), L and N (Jin et al., 2015). The use of hypervariable non coding regions (NCR) as additional molecular markers was proposed by different authors (Gavilán et al., 2018; Bodewes et al., 2020; Lam et al., 2020).

Mumps vaccination was introduced into childhood European Immunization Programs as part of the measles, mumps and rubella vaccine (MMR) during the 80s decade of the 20^th^ century. Particularly, MMR was introduced into the Spanish vaccination schedule in 1981 [Centro Nacional de Epidemiología, CIBERESP, ISCIII (2023)], and into the Dutch National Immunization Program in 1987 (van den Hof et al., 2003). In both countries, the incidence of mumps rapidly decreased after achieving high vaccination coverages. In Spain, the incidence dropped from 211/100,000 inhabitants in 1982 to 35/100,000 inhabitants in 1991 falling to 3.5/100,000 in 2004 (López-Perea et al., 2017). In 2005, the formerly dominant genotype H was replaced by genotype G (Echevarría et al., 2010). From this year to 2019, in Spain three different epidemic waves of MuV genotype G were observed with 5–6 years of periodicity (López-Perea et al., 2017). The last one started in 2015 and was abruptly interrupted in March 2020 by the severe measures adopted for the control of the COVID-19 pandemic [Centro Nacional de Epidemiología, CIBERESP, ISCIII (2023)]. In The Netherlands, major outbreaks were reported among vaccinated students from 2009 to 2012 (Sane et al., 2014), while from 2013 to 2020 only small local outbreaks and individual cases were reported (National Institute for Public Health, 2022). Similarly, MuV genotype G outbreaks were also detected among highly vaccinated populations in other countries (Brockhoff et al., 2010; Whelan et al., 2010; Greenland et al., 2012; Sane et al., 2014; Gavilán et al., 2022). Waning of vaccine-induced immunity (Lam et al., 2020), incomplete genotype cross-reactivity (Nö Jd et al., 2001) and antigenic drift (Šantak et al., 2013) were suggested as explanations.

Although many articles reporting MuV strains causing outbreaks in different individual countries were published in recent years (Braeye et al., 2014; Nedeljković et al., 2015; Park, 2015; Indenbaum et al., 2017; Willocks et al., 2017; Veneti et al., 2018; Moghe et al., 2019; el Zarif et al., 2020), studies at larger geographic scales are absent. The aim of the present study was to compare the circulation patterns of MuV in Spain and The Netherlands in a period of 5 years, as a step forward toward the study of broader patterns at European level.

## 2. Materials and methods

### 2.1. Samples and study period

A total of 188 Dutch and 933 Spanish sequences of the SH gene obtained from MuV genotype G cases deposited in GenBank as part of studies previously published by the authors of this study (Gavilán et al., 2018; Bodewes et al., 2020; Shah et al., 2021) were investigated. A subset of 262 MuV were selected for sequencing of the NCR region between the M and F protein (MF-NCR) from associated samples. MF-NCR sequences were collected from 127 MuV cases detected in the Netherlands, while 135 were detected in Spain. Samples were selected to be representative of the spatiotemporal distribution of mumps cases in both countries during the period of study, based on previous studies (Bodewes et al., 2020; Gavilán et al., 2022) and according to the availability of the samples. At least one case was selected for each location. When more than one case from a given location were available, they were selected to be representative of the whole period of circulation of the virus.

### 2.2. Nucleic acid extraction, genetic amplification and sequencing

For MuV detected from mumps cases in the Netherlands nucleic acids were extracted and the SH and MF-NCR region were amplified as described previously (Bodewes et al., 2020). sequences PCR-amplified products were purified with ExoSAP-IT (GE Healthcare). Sanger sequencing was subsequently performed at BaseClear (Leiden, the Netherlands). Spanish MuV cases were amplified according to published protocol (Royuela et al., 2011). Positive samples were purified by an enzymatic reaction using Illustra ExoProStar 1-Step (GE Health Care Life Science, Freiburg, Germany) and sequenced using the Sanger method with the ABI Big Dye Terminator Cycle Sequencing Kit (Applied Biosystems, Branchburg, NJ) using the corresponding forward and reverse primers (Gavilán et al., 2022).

### 2.3. Genetic and phylogenetic analysis and variant classification

**Figure 1:**
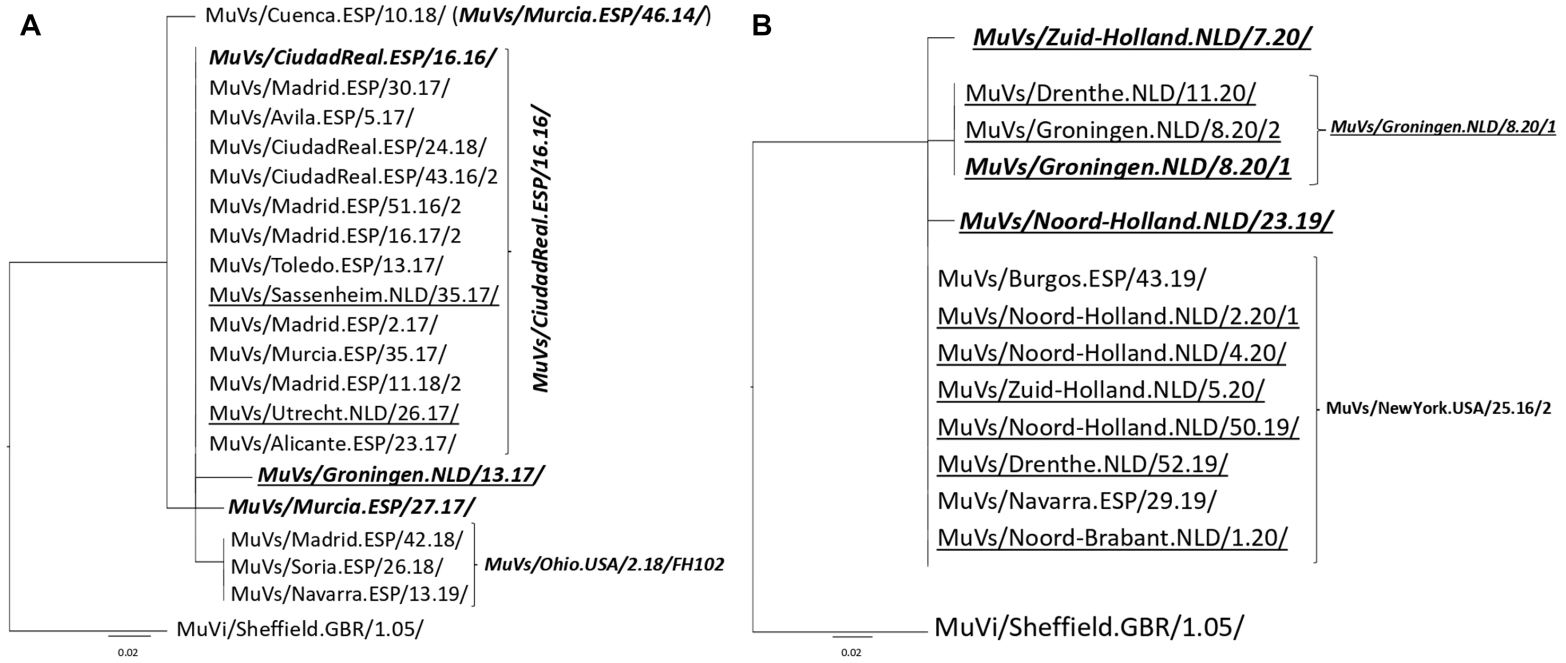
Phylogenetic analysis of SH-MF-NCR concatenated sequences obtained from SH variants. **(A)** MuVs/NewYork.USA/45.15/ SH variant analysis. **(B)** MuVs/Tarragona.ESP/20.11/ SH variant analysis. Phylogenetic trees were made using the maximum likelihood method in W-IQ-TREE, using HKY85 as substitution model. MuVi/Sheffield.GBR/1.05/ (ON148331) was used as outgroup. Underlined names correspond to Dutch concatenated sequences and italics bold names indicate the MF-NCR haplotype names.

**Figure 2:**
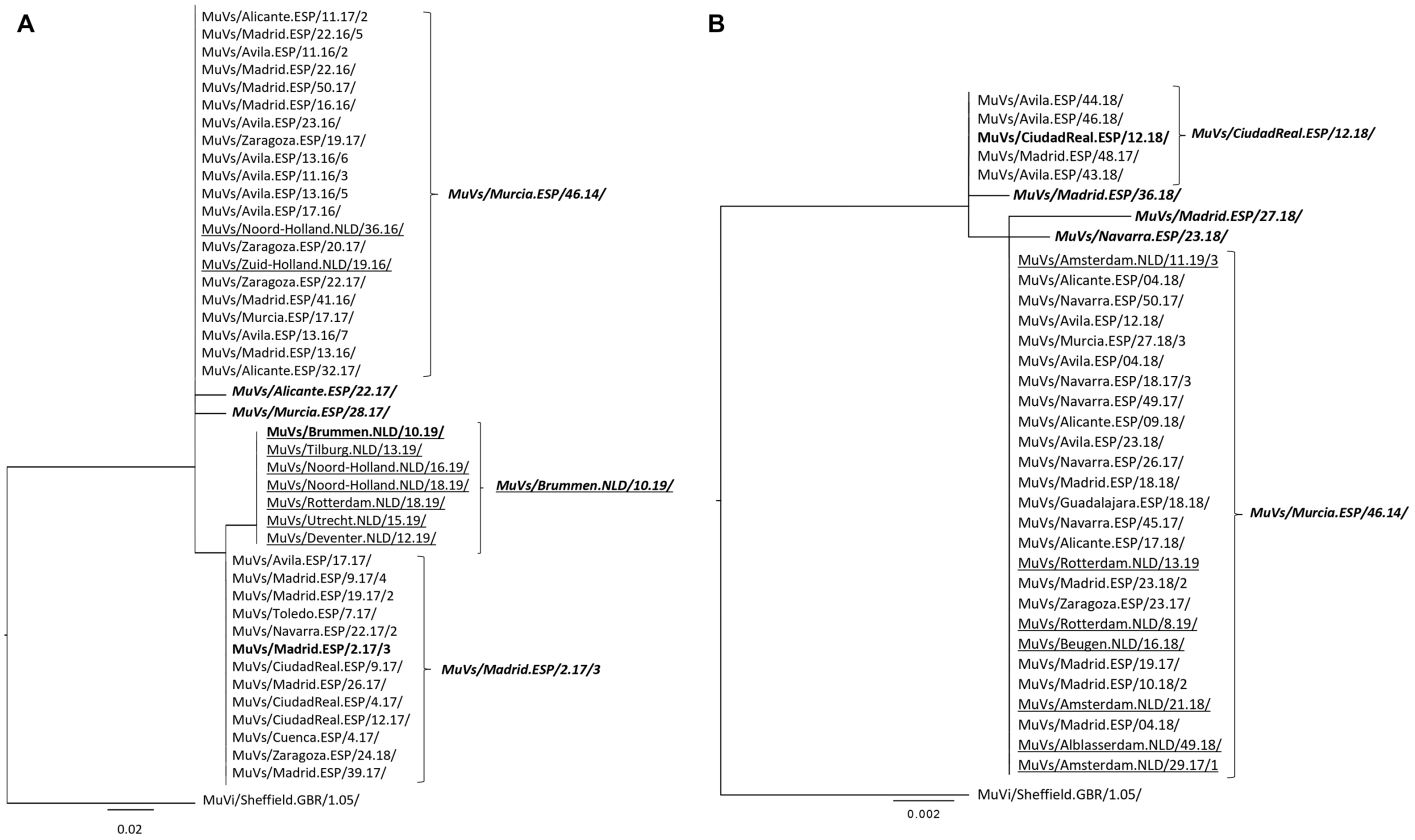
Phylogenetic analysis of SH-MF-NCR concatenated sequences obtained from SH variants. **(A)** MuVs/Avila.ESP/11.16/ SH variant analysis. **(B)** MuVs/Madrid.ESP/50.16/2 SH variant analysis. Phylogenetic trees were made using the maximum likelihood method in W-IQ-TREE, using TN93 as substitution model. MuVi/Sheffield.GBR/1.05/ (ON148331) was used as outgroup. Underlined names correspond to Dutch sequences. Italics bold names indicate the MF-NCR haplotypes.

**Figure 3:**
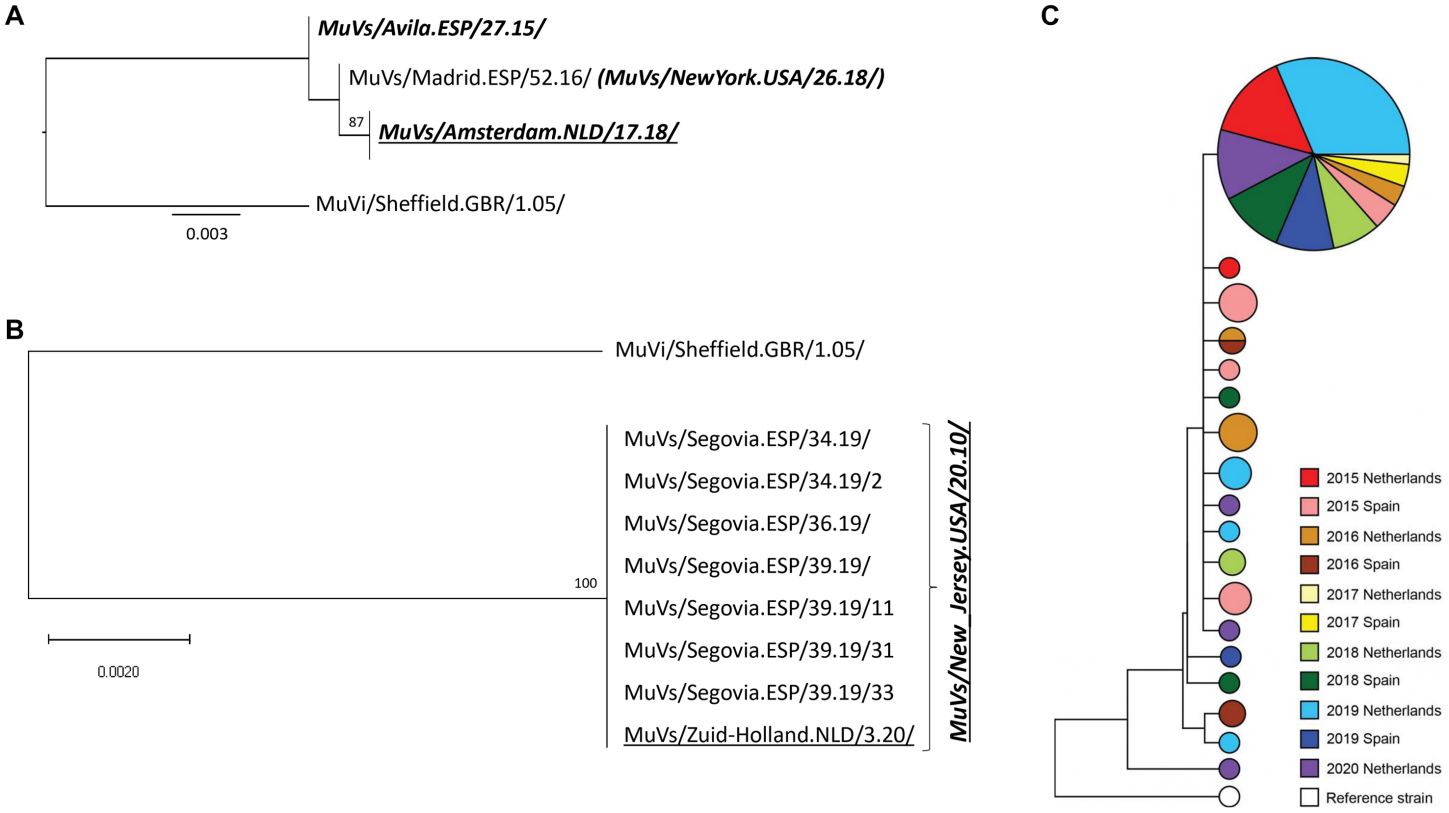
Phylogenetic analysis of SH-MF-NCR concatenated sequences obtained from SH variants. **(A)** MuVs/Minnesota.USA/53.14/ SH variant analysis. **(B)** MuVs/NewJersey.USA/20.11/ SH variant phylogenetic tree. **(C)** MuVi/Sheffield.GBR/1.05/ SH variant analysis. Panel A and B: phylogenetic trees were made using the maximum likelihood method in W-IQ-TREE, using HKY85 as substitution model. MuVi/Sheffield.GBR/1.05/ original sequence (ON148331) was used as outgroup. Underlined names correspond to Dutch sequences. Italics bold names indicate the MF-NCR haplotypes. **(C)** Phylogenetic tree was made using BioNumerics versión 7.6.3. Years are represented by different colors; Dutch concatenated sequences are represented by light color and Spanish concatenated sequences by darker color. Circle sizes are according to number of sequences.

All MuV sequences were edited and concatenated using Bioedit v.7.2.5 (Hall, 1999) and aligned using MAFFT v.7 (Katoh and Standley, 2013). Phylogenetic analysis was performed by the maximum likelihood method (ML) with IQ-TREE software *via* the webserver (W-IQ-TREE) (Trifinopoulos et al., 2016). Branch support was calculated using the ultrafast bootstrap approach (UFboot) (Hoang et al., 2018). Phylogenetic trees were edited using Figtree v.1.4.4.^1^ UltrafastBootstrap values were shown when they were higher than 80. The MuVi/Sheffield.GBR/1.05/ SH variant phylogenetic tree was built using concatenated sequences with UPGMA (unweighted pair group method with arithmetic mean) and 1000 bootstrap replicates using BioNumerics version 7.6.3 (2016).^2^ Individual phylogenetic trees for each SH-variant (see below) are displayed in Figures 1–3 as indicated in the footnotes.

The haplotype and variant definitions were used as described previously (Gavilán et al., 2022) with slight modifications. Briefly, a haplotype is a set of identical sequences named with the name of the earliest detected MuV according to WHO nomenclature. Haplotypes defined by SH sequence that showed an extensive circulation were considered variants. Extensive circulation is defined as continuous detection for 6 months or more and/or spreading to 3 or more provinces or different countries. Haplotype names are preceded by the suffix SH or MF-NCR to indicate the sequence fragment from which they are derived.

### 2.4. Ethics statement

All the Spanish samples used in this study were collected in the context of the Mumps Microbiological Surveillance Programme of the CNM^3^ and used in accordance with the requirements of Spanish biomedical research law (Ley 14/2007 de Investigación Biomédica). The protocol was approved by the Comité de Ética de la Investigación del Instituto de Salud Carlos III (approval no. Reference code: CEI PI 35-2015).

Molecular surveillance of mumps viruses, including this study, is part of the Public Health Act (BWBR0024705) in The Netherlands.^4^ Therefore, no informed consent was required for this study using anonymized routine surveillance data.

## 3. Results

### 3.1. Analysis of SH sequences haplotypes and variants

Analysis of SH sequences from The Netherlands and Spain revealed 106 different haplotypes during the period of study (Supplementary Figure 1). Of them, seven were considered SH variants according to the aforementioned criteria, namely MuVs/Tarragona.ESP/20.11/, MuVs/New_Jersey.USA/20.10/, MuVs/NewYork.USA/45.15/, MuVs/Avila.ESP/11.16/, MuVs/Madrid.ESP/50.16/2, MuVi/Sheffield.GBR/1.05. and MuVs/Minnesota.USA/53.14/ (Supplementary Figure 1; Supplementary Table 1). Circulation periods of each variant were always coincidental in both countries, although they were all detected first in Spain.

### 3.2. Circulation of haplotypes and variants

To increase the molecular resolution of the phylogenetic analysis of MuV variants and haplotypes detected in both Spain and The Netherlands, SH sequences were concatenated with MF-NCR sequences.

A single MF-NCR haplotype (MuVs/Murcia.ESP/46.14/) was present in 156 sequences (59.3%), and was shared by five of seven SH variants, being only absent from MuVs/Tarragona.ESP/20.11/ and MuVs/Minnesota.ESP/53.14/ SH variants. Only two sequences of MuVs/Zuid-Holland.NLD/5.20/ MF-NCR haplotype were shared by MuVs/Tarragona.ESP/20.11/ and MuVi/Sheffield.GBR/1.05/ SH variants (one each). The remaining 35 MF-NCR haplotypes were detected only in combination with a single SH variant. MuVs/Murcia.ESP/46.14/ MF-NCR haplotype was also present in both countries during the period of study. Also MuVs/Navarra.ESP/29.19/ MF-NCR haplotype was detected in both countries in 2019, and MuVs/Madrid.ESP/3.16/ MF-NCR haplotype in 2016 and MuVs/CiudadReal.ESP/16.16/ MF-NCR haplotype in 2017. In concordance with SH variants, all shared MF-NCR haplotypes were detected first in Spain. Only three MF-NCR sequences belonged to MuVs/Minnesota.ESP/53.14/ SH variant. Two of them were detected in Spain and one in The Netherlands. All of them share a characteristic nucleotide variation (C364T). Only MuVs/Murcia.ESP/46.14/ MF-NCR haplotype was detected in combination with the MuVs/New_Jersey.USA/20.10/ SH variant.

Of the five MF-NCR haplotypes derived from MuVs/NewYork.USA/45.15/ SH variant, only MuVs/Murcia.ESP/46.14/ MF-NCR haplotype and MuVs/CiudadReal.ESP/16.16/ MF-NCR haplotype were detected in both countries (Figure 1A). Two of the four remaining MF-NCR haplotypes were detected only in Spain and one only in The Netherlands. Interestingly, all five MF-NCR haplotypes shared the same nucleotide variant (A66C, Supplementary Table 1), except MuVs/Murcia.ESP/46.14/ MF-NCR haplotype, which appear as the most basal in the tree. MuVs/Murcia.ESP/46.14/ MF-NCR haplotype was not associated with MuVs/Tarragona.ESP/20.11/ SH variant (Figure 1B). All five MF-NCR haplotypes associated to this SH variant share the variant C352T. Four of them were present in The Netherlands and only one in Spain, which was, however, detected first. MuVs/Avila.ESP/11.16/ SH variant emerges associated to MuVs/Murcia.ESP/46.14/ MF-NCR haplotype on week 11 of 2016 in Spain and was detected sporadically in The Netherlands. On January of 2017 the change C60T was first detected in Spain (MuVs/Madrid.ESP/2.17/3 MF-NCR haplotype), which maintained parallel circulation with MuVs/Murcia.ESP/46.14/ MF-NCR haplotype in Spain. In 2019 (weeks 10–19) another MF-NCR haplotype with an additional change C313T (MuVs/Brummen.NLD/10.19/) was detected in The Netherlands, months after the last time MuVs/Avila.ESP/11.16/ SH variant was detected in Spain (Figure 2A). Five different MF-NCR haplotypes associated to MuVs/Madrid.ESP/50.16/2 SH variant were detected in Spain, together with MuVs/Murcia.ESP/46.14/ MF-NCR haplotype, which was the only one detected in The Netherlands (Figure 2B).

The only MF-NCR haplotype associated to the MuVs/New_Jersey.USA/20.10/ SH variant was MuVs/Murcia.ESP/46.14/ MF-NCR haplotype (Figure 3B). Only one of 252 cases caused by this SH variant was detected in The Netherlands. This case was imported from Spain according to the epidemiological data. The MuVi/Sheffield.GBR/1.05/ SH variant was detected in both countries during the complete study period with the exception of time range mid-2016 to mid-2017 in both countries. Only MuVs/Murcia.ESP/46.14/ MF-NCR haplotype was detected in both countries, among a total of 17 haplotypes associated to this variant.

## 4. Discussion

In the present study, sequence data from MuV detected in Spain and The Netherlands were analyzed to gain insights in the spatiotemporal spread of MuV at a larger geographical scale than in previous local studies. During the period of study seven SH variants circulated in both countries. All of them have been described in previous works (Bodewes et al., 2020; Shah et al., 2021; Gavilán et al., 2022).

Analysis of MF-NCR and SH concatenated sequences in Spain and The Netherlands provided novel insights into mumps virus circulation improving the analysis of SH gene recommended by WHO for MuV genotyping. Molecular resolution can be improved by the use of additional NCRs as described previously (Gavilán et al., 2018). However, the added value is relatively limited (Gavilán et al., 2018). Furthermore, analysis of complete genomes is a step forward on the way to understand MuV circulation patterns (Bodewes et al., 2020; Lam et al., 2020). However, full genome sequencing of mumps viruses is only available in a few countries and the use of subgenomic markers obtained with classical methods will continue to play a role for mumps surveillance for some time.

Analysis of MF-NCR sequences revealed a major haplotype, MuVs/Murcia.ESP/46.14/, which was first detected associated with MuVi/Sheffield.GBR/1.05/ SH variant in late 2014 in Spain (Gavilán et al., 2018). This haplotype was associated with five of six subsequent SH variants in both countries along the period of study. Specific MF-NCR haplotypes associated to certain SH variants seemed to emerge in each country after the spread of the MuVs/Murcia.ESP/46.14/ MF-NCR haplotype. Moreover, three other minor MF-NCR haplotypes were also shared in both countries: MuVs/Navarra.ESP/29.19/, MuVs/Madrid.ESP/3.16/ and MuVs/CiudadReal.ESP/16.16/. Interestingly, all of them were firstly detected in Spain as well as MuVs/Murcia.ESP/46.14/ MF-NCR haplotype. These results indicate a direction of spread from Spain to The Netherlands, which would be in agreement with the higher incidence rate of mumps in Spain in spite of similar immunization coverage. According to EUROSTAT, more than two million Dutch individuals visited Spain and more than three hundred thousand Spaniards traveled to The Netherlands each year before the COVID-19 pandemic. The exception to this model would be MuVs/Tarragona.ESP/20.11/ and MuVs/Minnesota.USA/45.15/ SH variants which were never detected associated with the MuVs/Murcia.ESP/46.14/ MF-NCR haplotype suggesting a different origin. In fact, results of our study suggest that MuVs/Minnesota.USA/45.15/ SH variant was not continuously circulating in Europe, since it was only sporadically detected during the study period.

The COVID-19 pandemic had a high impact on MuV circulation. The number of reported mumps cases in The Netherlands, Spain and various other European countries decreased sharply after extraordinary public health measures were implemented and is in spring 2023 still low. Of interest, the two first MuV genotype G SH haplotypes detected in The Netherlands in 2022 (MuVs/Zuid-Holland.NLD/20.22/; ON792317) and in Spain in 2023 (MuVs/Madrid.ESP/2.23/; OQ555726) had different SH sequences that grouped in different phylogenetic clade those described in this study (data not shown).

In conclusion, the present study provided novel insights into the circulation of MuV variants and haplotypes beyond the borders of single countries. In fact, the use of the MF-NCR molecular tool allows to reveal MuV spread between The Netherlands and Spain. Similar studies including other (European) countries are needed to provide a wider scope to the data shown in the present study.

## Data availability statement

The datasets presented in this study can be found in online repositories. The names of the repository/repositories and accession number(s) can be found in the article/Supplementary material.

## Ethics statement

The studies involving human participants were reviewed and approved by Comité de Ética de la Investigación (CEI) del Instituto de Salud Carlos III (ISCIII). Written informed consent for participation was not required for this study in accordance with the national legislation and the institutional requirements.

## Author contributions

AG: technical work, data analysis, and writing as main author. LN-R and AC: technical work. TW, NL-P, and JM-C: review and assistance in editing the final version of the manuscript. JE, AF-G, and RB: design for the study, data analysis, drafted, review and assistance in the editing of the final version of the manuscript.

## Funding

AG was funded by CIBER de Epidemiología y Salud Pública (CIBERESP), ISCIII. This work was supported by the “Instituto de Salud Carlos III” (PI15CIII/00023, PI19ICIII/0041).

## Conflict of interest

The authors declare that the research was conducted in the absence of any commercial or financial relationships that could be construed as a potential conflict of interest.

## Publisher’s note

All claims expressed in this article are solely those of the authors and do not necessarily represent those of their affiliated organizations, or those of the publisher, the editors and the reviewers. Any product that may be evaluated in this article, or claim that may be made by its manufacturer, is not guaranteed or endorsed by the publisher.

## Footnotes

^1^ http://tree.bio.ed.ac.uk/software/figtree/

^2^Applied Maths NV. Aavailable at: http://www.applied-maths.com/bionumerics

^3^ http://gesdoc.isciii.es/gesdoccontroller?action=download&id=08/02/2022-fc27d35665

^4^ https://wetten.overheid.nl/BWBR0024705/2021-02-22/0/informatie

## Supplementary material

SUPPLEMENTARY FIGURE 1 Phylogenetic analysis of SH variants and haplotypes. The phylogenetic tree was made using the maximum likelihood method in W-IQ-TREE, using HKY85 as substitution model. The MuV genotype G reference sequence used as outgroup was MuVi/Gloucester.GBR/32.96/ [G] (AF280799). Common variants in both countries are marked by blue squares. Dutch sequences are indicated by blue arrows.

SUPPLEMENTARY TABLE 1 Nucleotide differences of MF-NCR haplotypes. Data are shown according to the temporal and geographical distribution. MF-NCR nucleotide differences were identified using MuVi/Sheffield.GBR/1.05/ as reference (ON148331). Sequence name and MF-NCR haplotype name (name of the earliest available sequence) are indicated in two different columns.

SUPPLEMENTARY TABLE 2 GenBank accession numbers of MuV SH and MF-NCR sequences.
