# Supplementary figures and images for: Comparison of circulation patterns of mumps virus in the Netherlands and Spain (2015–2020)
Source: Front Microbiol. 2023 Jun 16;14:1207500. doi: 10.3389/fmicb.2023.1207500 (PMC10311905; doi:10.3389/fmicb.2023.1207500)

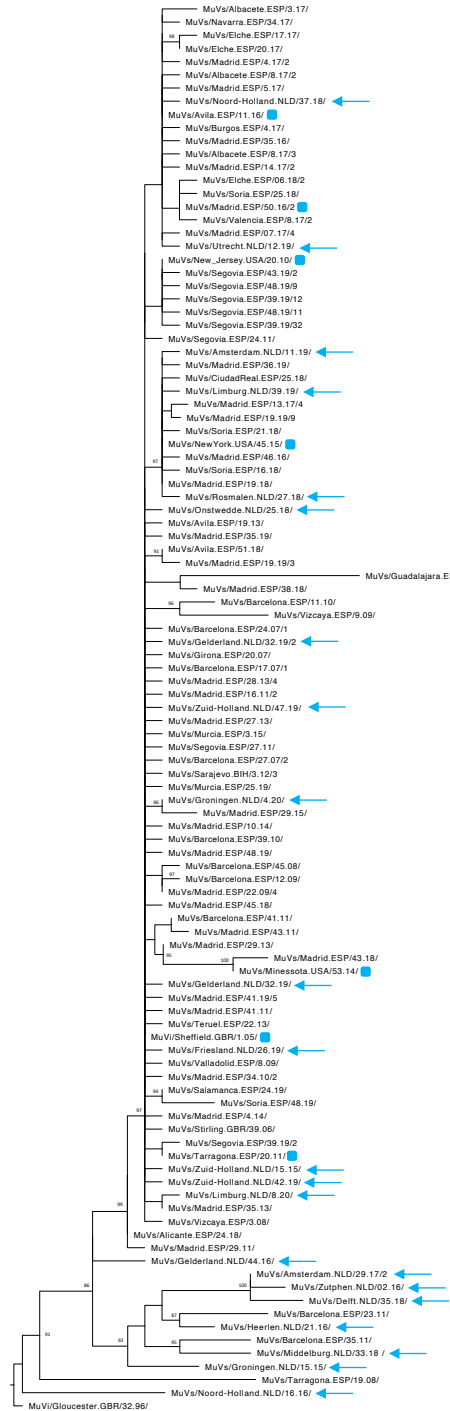

Supplement: SUPPLEMENTARY FIGURE 1 — Phylogenetic analysis of SH variants and haplotypes. The phylogenetic tree was made using the maximum likelihood method in W-IQ-TREE, using HKY85 as substitution model. The MuV genotype G reference sequence used as outgroup was MuVi/Gloucester.GBR/32.96/ [G] (AF280799). Common variants in both countries are marked by blue squares. Dutch sequences are indicated by blue arrows. [file Data_Sheet_1.zip › Supplementary Figure 1.pdf]
